# Status of Analgesic Drugs and Quality of Life Results for Diabetic Peripheral Neuropathy in China

**DOI:** 10.3389/fendo.2021.813210

**Published:** 2022-01-21

**Authors:** Jingxuan Lian, Haijun Wang, Rongrong Cui, Chaoxia Zhang, Jianfang Fu

**Affiliations:** ^1^ Department of Endocrinology, Xijing Hospital, The Air Force Medical University, Xi’an, China; ^2^ Department of Endocrinology, Yan’an People’s Hospital, Yan’an, China; ^3^ Department of Endocrinology, Shangluo Central Hospital, Shangluo, China; ^4^ Department of Endocrinology, Xi’an Daxing Hospital, Xi’an, China

**Keywords:** diabetic peripheral neuropathy, NRS, PHQ-9, GAD-7, analgesics

## Abstract

**Objective:**

The purpose of this study is to describe the current clinical situation of patients with painful diabetic peripheral neuropathy (DPN) and related anxiety, depression, and the quality of life of patients in mainland China, and to report the current status of the use of analgesics.

**Methods:**

Between June 15, 2021, and October 15, 2021, a total of 401 participants participated in the study. Recruitment was carried out using a multi-level sampling method. Participants’ demographics, medical history, analgesic use, Michigan Symptom Score (MNSI), Numerical Rating Scale (NRS) pain score, Patient Health Questionnaire 9 (PHQ-9) score, Generalized Anxiety Disorder 7 (GAD) -7) Score, quality of life score (SF-12) and diabetes treatment status were collected.

**Results:**

Among the participants, there were 236 male patients and female patients. Participants were 322 patients over 40 years old. Regarding the use of analgesics: 132 patients reported using analgesics, 221 patients reported not using analgesics, and 48 patients reported having used analgesics. The results of the scale showed that the scores of NRS, GAD-7, PHQ-9 and SF-12 were 5.12 ± 2.15, 6.33 ± 3.67, 8.46 ± 4.07 and 47.84 ± 19.92 for patients who used analgesics, Compared with patients who did not use analgesics (NRS: 1.99 ± 1.7, GAD-7: 1.81 ± 2.81, PHQ-9: 3.13 ± 4.10, SF-12: 78.34 ± 21.66) there are significant differences (p< 0.001). In addition, patients’ NRS scores are also closely related to GAD-7, PHQ-9 and SF-12 scores.

**Conclusion:**

The severity of symptoms, mental status and quality of life of patients who used analgesics were more severe than those of patients who did not use analgesics. Pregabalin is still the preferred analgesic for patients with painful DPN, and the use of opioids in my country is extremely low, which is consistent with current international guidelines. Age, diabetic duration, DPN duration, PHQ-9 score, GAD-7 score and SF-12 scores are closely related to NRS pain scores. In addition, there are still a considerable number of patients who have not used analgesics due to financial burdens and other reasons, suggesting that China still has insufficient pain management in DPN patients.

## Introduction

Diabetic Peripheral Neuropathy (DPN) is one of the important complications of type 2 diabetes and the most common cause of neuropathy in developed countries. It is estimated that about 50% of diabetic patients are affected; its most common form It is chronic, distal and symmetric sensorimotor polyneuropathy. Other uncommon forms include asymmetric or focal neuropathy, such as diabetic muscle atrophy, trunk radiculopathy, and compression paralysis (1, 2). The onset of DPN is insidious and is a chronic clinical process. It is likely to develop into foot deformities, foot ulcers, and severe cases can lead to amputation (3).

Studies have shown that about 20-50% of diabetic patients and about 60% of DPN patients will present with painful neuropathy. However, the specific mechanism of DPN is still unclear. Current studies believe that the changes of microvascular, such as the abnormal structure and function of blood vessels and nerves and the adjustment of peripheral blood flow are closely related to painful DPN; coupled with the release of oxidative stress and inflammatory factors, the combined action of the three ultimately leads to peripheral The changes in nerve structure and function eventually lead to painful DPN (4–6). Clinically, painful DPN is mainly manifested as loss of skin sensation and pain in the distal extremities, of which symmetrical pain in the lower extremities is the most common (7). Other manifestations include atypical pain, numbness, acupuncture and burning sensation, and can also be manifested as other neuromotor dysfunctions, such as muscle weakness, poor balance, and tendency to fall (8). Studies have shown that up to 60% of diabetic patients are deeply affected by painful diabetes, leading to the occurrence of serious physical and social diseases, and seriously affecting the quality of life of patients (9). Patients with painful DPN have anxiety, depression and sleep disorders. And other mental problems, even the 10-year mortality rate of patients with painful DPN is higher than that of patients with non-painful DPN. Painful DPN will also bring a serious economic burden to society and families. It is estimated that the annual treatment cost of each DPN patient is twice that of diabetic patients, while the cost of moderate to severe pain patients is 4 times that of non-pain DPN patients. Times. The management of painful DPN changes remains a challenge in the clinic (10).

Therefore, this study conducted a cross-sectional study on the use of analgesics in patients with painful DPN, the symptoms, mental status, and quality of life in patients with painful DPN through questionnaire surveys. So as to help doctors to choose the painful DPN analgesic plan reasonably in the clinic.

## Method

### Study Designs and Participants

This multi-center cross-sectional study was approved by Xijing Hospital of the Air Force Military Medical University in March 2021. All Participants were recruited from the Endocrinology Department of 8 hospitals in Shaanxi Province, starting on March 1, 2021, and ending on September 30, 2021.

Inclusion criteria was: (1) Age over 18; (2) A clear diagnosis of type 1 or type 2 diabetes; (3) The patient has clear symptoms, signs or electrophysiological evidence of DPN; (4) The patient complains of clear spontaneous pain (continuous or intermittent) Acupuncture, electric shock, burning pain, etc.) or induced pain (hyperalgesia and allodynia); (5) Chronic pain for at least 3 months; (6) Sign informed consent. The exclusion criteria was: (1) Patients without DPN, or combined with nociceptive or mixed pain (such as cervical/lumbar spine), neuralgia after herpes zoster, arthritis, spinal cord disease and other diseases that may cause peripheral neuropathic pain (Immunity, toxicity, nutritional neuropathy, etc.); (2) Dementia, drug abuse or other conditions that seriously impair cognition and communication; (3) The patient does not agree to participate in the survey. Participants were given the opportunity to complete the survey questionnaires either online or in paper form. Study participation was entirely voluntary, and no financial incentives were offered.

Sample size was determined using a single population proportion formula with the assumptions of a 5% level of significance (95% confidence interval [CI]), a 4% margin of error (d). Due to the large differences in medical resources and clinical diagnosis and treatment levels in different hospitals and regions, this will result in a certain difference between the treatment plans of different hospitals and the current guidelines. In addition, according to the relevant literature (11), we assume that 30% of patients are considered to have received first-line drug treatment that does not meet the guidelines recommended. In this case, to obtain an accurate non-conformity rate of 5%, that is, a 95% CI between 25% and 35% (with a bilateral width of 10%), a total of 362 patients were required. Considering the differences in record compliance between the centers and other reasons, we expanded the required sample size by 10%, and finally required 398 DPN patients in China. The required sample size is calculated by PASS V.14.

### Quality Control of the Questionnaire

This study uses a cluster sampling method. The endocrinology department of each hospital has a trained attending doctor as an investigator. The similarity of the number of people sampled and the number of working days in each center is a prerequisite for ensuring the representativeness of the population of the sampling organization. To ensure that the number of working days drawn by all centers is similar, the task of recruiting patients is mainly divided into three stages, and the initial stage is gradually adjusted according to the recruitment progress. The first stage is the running-in period, and the enrollment of patients is slower. The first phase will last for 1 week. During this period, each center will choose 1.5 outpatient days per week. The number of working days required for subsequent actual recruitment is mainly estimated based on the number of eligible patients per day in each center in the first phase. In the second stage, the collection rate was adjusted to 4 outpatient days per week. In the third stage, after 250 cases have been collected, the arrangement of subsequent collection days will be adjusted according to the actual number of completions in each center to ensure that the working days for the number of people collected by each center are the same. The Xijing Hospital Review Committee reviewed and approved the study (XJYY-LL-WJ-272), and all participants in the study provided informed consent.

### Measures

#### Pain and DPN Symptoms

Using Michigan Neuropathy Screening Instrument (MNSI) (12)questionnaire scores to assess the patient’s symptoms. Numerical Rating Scale was used to assess the patient’s pain level which provides 0–10 scale reflecting the no pain and extreme pain.

#### Quality of Life

Two instruments were used to assess carers’ QoL: (1) Using a visual analogue scale (VAS) that provides a single global assessment of self-reported health on a 0–100 scale reflecting the worst and the best imaginable health state. (2) The 12-item Short Form Health Survey 12v2 (SF-12) from General health (GH), physical function (PF), Physical function (RP), physical pain (BP), mental health (MH), vitality (VT), social Function (SF), and emotional function (RE) 8 dimensions are used to measure the mental and physical health of patients.

#### Depression and Anxiety

PHQ-9 is used to measure depression symptoms. PHQ-9 is a 9-item questionnaire that determines the severity of depression by asking participants about the degree of distress on the 9 criteria of depression symptoms ranging from 0 (not at all) to 3 (almost every day). day). The scoring is additive ranging from 0 to 27, with higher scores indicating greater severity of depression. The Generalized Anxiety Disorder scale (GAD-7) was applied to obtain the anxious status. The scoring is additive ranging from 0 to 21, with higher scores indicating greater severity of anxiety. Both scales have previously been established as a reliable and valid measure of depression severity.

#### Analgesics Provision

Informed by the published literature, a 5-point Likert scale was developed to measure the extent of support provided. A free-text box was included allowing participants to comment on how they feel about analgesics.

#### Statistical Analysis

linear regression was used to determine whether quality of life, depression and anxiety were associated with NRS. Characteristics that were significantly associated with NRS (age, sex, BMI, complication) were included as covariates in the final linear regression model. Using chi-square test, Wilcoxon Mann-Whitney test, or t test as appropriate for comparison between groups. p value <0.05 is considered statistically significant. The internal consistency of SF-12, PHQ-9 and GAD-7 scales was tested by Cronbach α coefficient, and the validity of the scales was analyzed by Barlett sphere test.

## Results

### Participant Characteristics

Demographic and clinical characteristics for participants included in this study are provided in **Table 1**. Of the 401 patients included in the cohort, 221 patients reported not using analgesics. 132 patients reported using analgesics, 48 patients reported having used analgesics. The age of the patients in this study cohort is mainly over 40 years old. The number of patients with DPN course of less than 10 years is 304 (75.81%). The overall patient education level is also low. The number of junior high school and high school education is as high as 271 (67.58%). The age of each group is, there was no significant difference between gender, DM and DPN course.

**Table 1:** Clinical and sociodemographic characteristics of patients with diabetic peripheral neuropathy in China.

Characteristic	All patients	Analgesic drugs: No	Analgesic drugs: Yes		
			Using	Had	Total
**n**	401	221	132	48	180
**Sex, n (%)**					
** Male**	236 (58.85)	136 (61.54)	69 (52.27)	31 (64.58)	100 (55.56)
** Female**	165 (41.15)	85 (38.46)	63 (47.73)	17 (35.42)	80 (44.44)
**Height, cm, mean (SD)**	166.33 (9.87)	167.32 (8.33)	164.29 (12.5)	167.38 (6.77)	165.11 (11.38)
**Weight, kg, mean (SD)**	70.04 (15.01)	71.76 (16.36)	67.65 (12.09)	68.46 (11.36)	68.09 (12.98)
**BMI, kg/m2, mean (SD)**	25.58 (10.01)	25.53 (4.97)	26.09 (16.03)	24.33 (2.96)	25.70 (12.93)
**Age, n (%)**					
** <25**	9 (0.75)	7 (3.17)	2 (1.52)	0 (0)	2 (1.11)
** 26~30**	18 (4.49)	15 (6.79)	1 (0.76)	2 (4.17)	3 (1.67)
** 31~40**	52 (12.97)	43 (19.46)	6 (4.55)	3 (6.25)	9 (5)
** 41~50**	99 (24.69)	57 (25.79)	27 (20.45)	15 (31.25)	42 (23.33)
** 51~60**	125 (31.17)	58 (26.24)	54 (40.91)	13 (27.08)	67 (37.22)
** >60**	98 (24.44)	41 (18.55)	42 (31.82)	15 (31.25)	57 (31.67)
**Duration of DM, n (%)**					
** <5**	122 (30.42)	103 (46.61)	11 (8.33)	8 (16.67)	19 (10.56)
** 6~10**	88 (21.95)	53 (23.98)	23 (17.42)	12 (25.00)	35 (19.44)
** 11~15**	85 (21.2)	27 (16.74)	31 (23.48)	17 (25.43)	48 (26.67)
** 16~20**	57 (14.21)	17 (7.69)	33 (25.00)	7 (14.58)	40 (22.22)
** >20**	49 (12.22)	11 (4.98)	34 (25.76)	4 (8.33)	38 (21.11)
**Duration of DPN, n (%)**					
** <5**	201 (50.12)	161 (72.85)	27 (20.45)	13 (27.08)	40 (22.22)
** 6~10**	103 (25.69)	34 (15.38)	46 (34.85)	23 (47.92)	69 (38.33)
** 11~15**	52 (12.97)	14 (6.33)	30 (22.73)	8 (16.67)	38 (21.11)
** >15**	45 (11.22)	12 (5.43)	29 (21.97)	4 (8.33)	33 (18.33)
**Education, n (%)**					
** Junior school and below**	142 (35.41)	76 (34.39)	54 (40.91)	12 (25.00)	56 (31.11)
** High school**	129 (32.17)	61 (26.60)	50 (37.88)	18 (37.50)	68 (37.78)
** Junior college**	69 (17.21)	38 (17.19)	21 (15.91)	10 (20.83)	31 (17.22)
** Undergraduate**	55 (13.72)	42 (19.00)	6 (4.55)	7 (14.58)	13 (7.22)
** Master and above**	6 (1.5)	4 (1.81)	1 (0.76)	1 (2.08)	2 (1.11)
**Treatments, n (%)**					
** None**	26 (6.48)	24 (10.86)	0 (0)	0 (0)	0 (0)
** Oral hypoglycemic**	217 (54.11)	125 (56.56)	65 (49.24)	29 (60.42)	94 (52.22)
** Insulin**	29 (7.23)	11 (4.98)	15 (11.36)	16 (33.33)	31 (17.22)
** Both oral and insulin**	129 (32.17)	61 (27.6)	52 (39.39)	3 (6.25)	55 (30.56)

BMI, body mass index; DM, diabetes mellitus; DPN, Diabetic peripheral neuropathy.

### Reliability and Validity Test of Scale

The Cronbach’s α obtained for the GAD-7’s seven items were an excellent value (0.867) and it maintains excellent even if we delete an item, as shown in the second column of **Table 2**. The Cronbach α of the SF-12 scale is 0.882 (**Table 3**). After deleting this dimension, it is above 0.85. The 8 dimensions of the scale were all positively correlated, and the correlation coefficients between each dimension are lower than the internal consistency coefficient of each dimension (**Table 3**). The Cronbach α of PHQ-9 is 0.891 (**Table 4**). In addition, we also performed a validity test. As shown in **Table 5**, the KMO of GAD-7, PHQ-9 and SF-12 are all greater than 0.85, and the Bartlett sphericity test is all p<0.001. The above results indicate that the three questionnaires are in This study has excellent validity and reliability.

**Table 2:** Cronbach α and correlation coefficient of different items on the GAD-7 scale.

Entry	Cronbach α If item deleted	GAD1	GAD2	GAD3	GAD4	GAD5	GAD6	GAD7
GAD1	0.839	1.000	0.565	0.532	0.496	0.472	0.483	0.566
GAD2	0.839	0.565	1.000	0.641	0.477	0.424	0.457	0.515
GAD3	0.841	0.532	0.641	1.000	0.521	0.430	0.411	0.491
GAD4	0.849	0.496	0.477	0.521	1.000	0.490	0.378	0.433
GAD5	0.853	0.472	0.424	0.430	0.490	1.000	0.486	0.374
GAD6	0.854	0.483	0.457	0.411	0.378	0.486	1.000	0.464
GAD7	0.849	0.566	0.515	0.491	0.433	0.374	0.464	1.000

Red means that the correlation coefficient is 0 and green is 1, and the color gradient of red to green means the transition of correlation from 0 to 1.

**Table 3:** SF-12 scale Cronbach α and correlation coefficient of different dimensions.

Dimension	Cronbach α If item deleted	PF	RP	BP	GH	VT	SF	RE	MH
**PF**	0.869	1.000	0.693	0.584	0.687	0.757	0.733	0.302	0.666
**RP**	0.875	0.693	1.000	0.632	0.665	0.661	0.732	0.396	0.689
**BP**	0.87	0.584	0.632	1.000	0.666	0.569	0.619	0.324	0.944
**GH**	0.87	0.687	0.665	0.666	1.000	0.634	0.699	0.355	0.759
**VT**	0.871	0.757	0.661	0.569	0.634	1.000	0.731	0.312	0.662
**SF**	0.868	0.733	0.732	0.619	0.699	0.731	1.000	0.357	0.698
**RE**	0.934	0.302	0.396	0.324	0.355	0.312	0.357	1.000	0.345
**MH**	0.858	0.666	0.689	0.944	0.759	0.662	0.698	0.345	1.000

Red means that the correlation coefficient is 0 and green is 1, and the color gradient of red to green means the transition of correlation from 0 to 1.

**Table 4:** PHQ-9 Cronbach α and correlation coefficient of different items.

Entry	Cronbach αIf item deleted	PHQ1	PHQ2	PHQ3	PHQ4	PHQ5	PHQ6	PHQ7	PHQ8	PHQ9
**PHQ1**	0.862	1.000	0.610	0.542	0.617	0.473	0.465	0.470	0.498	0.435
**PHQ2**	0.867	0.610	1.000	0.537	0.506	0.379	0.496	0.479	0.446	0.370
**PHQ3**	0.868	0.542	0.537	1.000	0.661	0.450	0.322	0.505	0.431	0.295
**PHQ4**	0.858	0.617	0.506	0.661	1.000	0.578	0.427	0.533	0.557	0.393
**PHQ5**	0.874	0.473	0.379	0.450	0.578	1.000	0.413	0.380	0.425	0.264
**PHQ6**	0.875	0.465	0.496	0.322	0.427	0.413	1.000	0.442	0.433	0.396
**PHQ7**	0.870	0.470	0.479	0.505	0.533	0.380	0.442	1.000	0.522	0.320
**PHQ8**	0.870	0.498	0.446	0.431	0.557	0.425	0.433	0.522	1.000	0.306
**PHQ9**	0.883	0.435	0.370	0.295	0.393	0.264	0.396	0.320	0.306	1.000

Red means that the correlation coefficient is 0 and green is 1, and the color gradient of red to green means the transition of correlation from 0 to 1.

**Table 5:** Validity test of GAD-7, PHQ-9 and SF-12.

Scale	KMO value	Bartlett sphericity test	
		Approximate chi-square	Significance
**GAD-7**	0.891	1106.195	**0.000**
**PHQ-9**	0.905	1529.923	**0.000**
**SF-12**	0.881	2836.494	**0.000**

Bold indicates p < 0.05.

### Pain and Psychological Status

**Figure 1:**
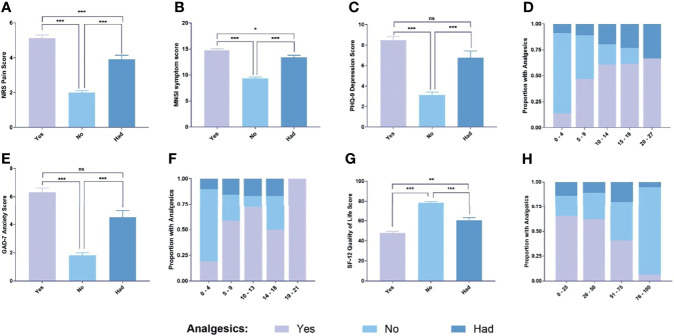
Different scale scores. **(A)** NRS scores for each group; **(B)** MNSI symptom score of each group; **(C)** PHQ-9 scores for each group; **(D)** Distribution of PHQ-9 scores in each group; **(E)** GAD-7 scores for each group; **(F)** Distribution of GAD-7 scores in each group; **(G)** SF-12 scores for each group; **(H)** Distribution of SF-12 scores in eachv group. Data are means ± SEM. ns, no significance. *P < 0.05, **P < 0.01 and ***P < 0.001.

As shown in **Figure 1A**, the NRS of patients who used analgesics was 5.12 ± 2.15, which was significantly different from the patients who did not use analgesics (1.99 ± 1.7, p <0.001). The NRS of patients who had used analgesics was 3.92 ± 1.60, which was statistically different from the above two groups. As for the Michigan symptom score (**Figure 1B**), the MISI score of patients who did not use analgesics (9.39 ± 3.29) was significantly lower than that of patients who used them(14.75 ± 3.50)(p < 0.001). In addition, it was also significantly lower than that of patients who had used them (13.38± 2,89)(p < 0.05). Although the patients who used analgesics were statistically significant compared to those who had used them, the difference in MNSI between the two was not clinically significant.

As shown in **Figure 1C**, in terms of depression and anxiety revealed by PHQ-9(8.46 ± 4.07 vs 3.13 ± 4.10, p < 0.001) and GAD-7(6.33 ± 3.67 vs 1.81 ± 2.81, p <0.01) (**Figure 1E**), the scores on GAD-7 and PHQ-9 scales of patients who used analgesics were significantly higher than those of non-use patients. And as shown in **Figures 1D, F**, as the GAD-7 and PHQ-9 scale scores increase, the proportion of patients using analgesics in the corresponding score segment also gradually increases. On the other hand, with the SF-12 scale, the higher the score, the higher the proportion of patients who did not use analgesics (**Figure 1H**), and the SF-12 score of patients who did not use analgesics was significantly higher than that of those who used analgesics (**Figure 1G**).

### Status of Analgesic

**Figure 2:**
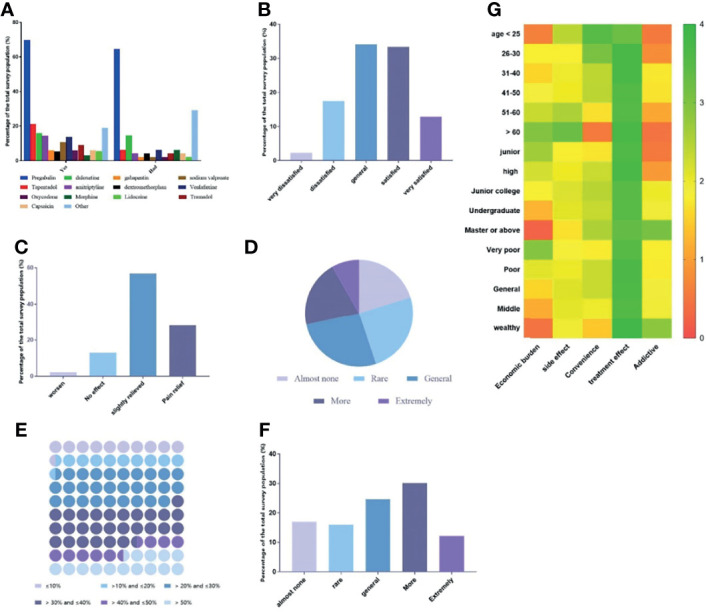
The current status of the use of analgesics in each group of patients. **(A)** Currently commonly used analgesics for DPN patients; **(B)** Patient satisfaction with analgesics; **(C)** Patient’s self-reported pain relief after medication; **(D)** Patients report the economic burden of using analgesics; **(E)** The ratio of the cost of analgesics to the cost of diabetes treatment; **(F)** Patient’s self-reporting of the effect of analgesics on the quality of life; **(G)** The patient’s preference for the considerations of choosing analgesic drugs.

We also investigated the specific situation of DPN patients using analgesics. As shown in **Figure 2A**, pregabalin is the first choice for patients who use and have used analgesics, followed by duloxetine and tapentadol. As for other drugs, it is mainly epalrestat. When asked whether they were satisfied with the efficacy of analgesics, only 46.18% of the patients were satisfied with the efficacy, and only 28.03% of the patients believed that the analgesics had significantly reduced pain, and 56.82% of patients believed that the effect was average, and even 2.27. % Of patients think that the pain has worsened. In addition, in terms of economic burden, 50.75% of patients spend more than 30% of the cost of analgesic drugs for diabetes treatment, and only 44.89% of people think that the economic burden of analgesics is relatively low. On the other hand, the side effects of analgesics also affect the daily life of patients. As shown in **Figure 2F**, 42.39% of patients believe that the side effects of analgesics affect their daily life and work.

The Likert 5-point scale starts from five aspects: economic burden, side effects, convenience, efficacy, and addiction, was developed to measure the main factors that patients consider to use analgesics. The main factors for patients to consider using analgesics were measured from the three dimensions of age, education level and income level, and a heat map was made. As shown in **Figure 2B**, there are significant differences in the main factors considering the use of analgesics for different ages, education and income levels (p <0.001). All patients regard the efficacy of analgesic drugs as the primary consideration, and secondly, only high-income groups pay more attention to drug addiction (2.86 ± 0.23). Other detailed results are shown in **Figure 2**.

### Association With NRS

**Figure 3:**
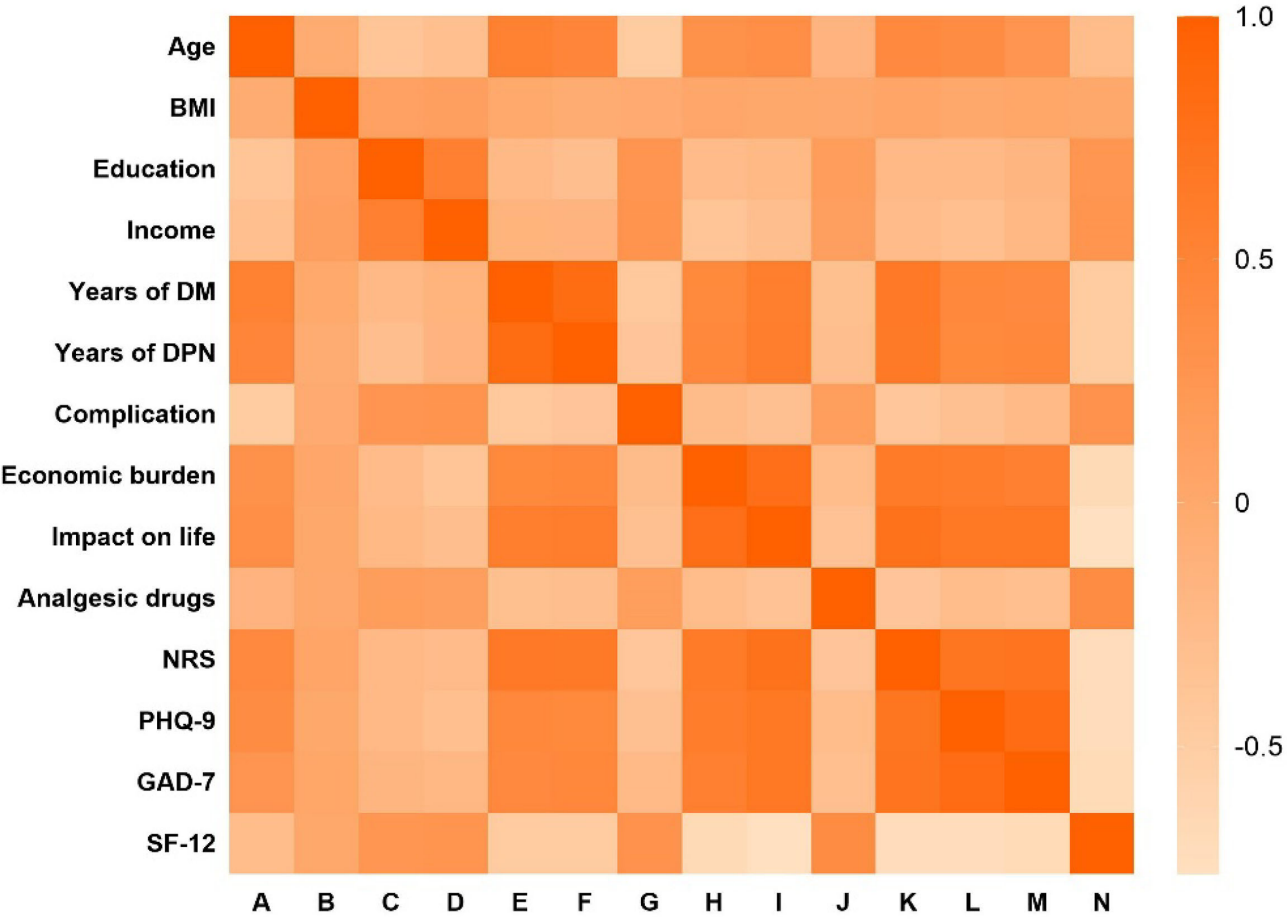
Heat map of the correlation between NRS scores and different factors.

To further study which factors are related to the NRS score of DPN patients, we conducted a correlation analysis. As shown in **Figure 3**, Pearson’s correlation analysis shows that NRS is closely related to GAD-7, SF-12, PHQ-9, the course of diabetes, and the course of DPN, and has low correlation with BMI, education, and income. Using a linear regression model, we evaluated whether NRS of DPN patients was associated with several quality of life, mental, and emotional measures. Patients with higher NRS reported significantly higher PHQ-9 depression score (covariate-adjusted β = 0.061, 95% CI: 0.011–0.111, p = 0.080) and GAD-7 anxiety score(covariate-adjusted β = 0.172, 95% CI: 0.11–0.234, p < 0.001) indicating worse mental health. Similarly, patients with higher NRS reported significantly lower SF-12 quality of life score (covariate-adjusted β = -0.047, 95% CI: -0.049– -0.044, p < 0.001) suggesting these patients’ overall health is worse than those who have lower NRS. Accordingly, all SF-12 sub-scores except SF and VT were significantly lower in DPN patients with low NRS than in controls (**Table 6**).

**Table 6:** Association between NRS and mood measures and SF-12 in DPN patients.

Clinical Characteristic	β (95% CI)	p-value	Covariate-Adjusted β (95% CI)	Covariate- Adjusted p-value
**PHQ-9**	0.068 (0.013,0.122)	**p = 0.150**	0.061 (0.014,0.111)	**p = 0.080**
**GAD-7**	0.178 (0.111,0.246)	**p < 0.001**	0.172 (0.110,234)	**p < 0.001**
**SF-12**	-0.044 (-0.052, -0.035)	**p < 0.001**	-0.047 (-0.049, -0.044)	**p < 0.001**
** PF**	-0.02 (-0.03, -0.009)	**p < 0.001**	-0.018 (-0.028, -0.010)	**p < 0.001**
** RP**	-0.014 (-0.027, -0.001)	**p = 0.035**	-0.012 (-0.022, -0.002)	**p = 0.021**
** BP**	0.019 (0.005,0.034)	**p = 0.009**	0.020 (0.006, 0.027)	**p < 0.001**
** GH**	-0.025 (-0.036, -0.014)	**p < 0.001**	-0.030 (-0.041, -0.020)	**p < 0.001**
** VT**	-0.005 (-0.014,0.004)	**p = 0.284**	-0.014 (-0.02, -0.008)	**p = 0.127**
** SF**	0.002 (-0.009,0.013)	**p = 0.685**	-0.009 (-0.034,0.016)	**p = 0.489**
** RE**	-0.01 (-0.023,0.003)	**p = 0.129**	-0.021 (-0.031,-0.011)	**p = 0.047**
** MH**	-0.044 (-0.061, -0.026)	**p < 0.001**	-0.065 (-0.078, -0.053)	**p < 0.001**

The higher scores for GAD-7 and PHQ-9 are indicative of more negative outcomes, while lower scores for SF-12 measures are indicative of more negative outcomes.

Covariates included in the linear regression included age at sex, BMI at enrollment and complication.

Bold indicates p < 0.05.

## Discussion

Due to the significant changes in economic growth, urbanization and lifestyles this year, the prevalence of diabetes in mainland China has increased rapidly. Inevitably, the prevalence of long-term complications will increase, which is costly for both the individual and the health system. The International Diabetes Federation predicts that the number of diabetic patients in China will be 116.4 million in 2019, which will increase to 140.5 million by 2030 and 147.2 million by 2045 (13, 14). The management of painful DPN will be a challenge due to the lack of unified early diagnosis criteria, blood glucose control and pain relief, and insufficient monitoring of comorbidities. To meet this challenge, we recently investigated the clinical symptoms, quality of life, mental health, and analgesic use of painful DPN.

We evaluate our findings as an effective elucidation of the clinical features and treatment of patients with painful DPN in mainland China. We found that in patients with painful DPN who used analgesics, the average pain severity NRS (± SD) score was 5.12 ± 2.15, and 80.8% of the cases reported moderate (NRS: 4-6) and severe pain (NRS: 7-10). This is slightly higher than other studies. According to reports, the average pain NRS score (± SD) of DPN in the Chinese study was 4.12 ± 2.07 (15). Scholar shows that the average pain severity score of DPN in the American study is 5.2 (0 -10 grade), and 79.5% of patients report moderate or severe pain (16). We have used MNSI, a straightforward and easy-to-use questionnaire which has been well-established and is a particularly amenable tool in our large population of painful DPN patients (17, 18). The results showed that the MNSI symptom score of DPN patients who used analgesics was much higher than that of patients who did not use analgesics. In addition, 52.3% of patients who used analgesics had a MNSI score greater than 14, while only 7.1% of patients who did not use analgesics had a MNSI score greater than 14, indicating that the severity of symptoms in patients who use analgesics is more serious than patients who do not use analgesics.

We also conduct a survey on the use of analgesics in patients with painful DPN. Our investigation found that pregabalin is still the drug of choice, 69.7% of patients are using pregabalin, followed by duloxetine, currently only these two drugs have been approved by the FDA and the European Medicines Agency for diabetes Neuropathic pain. In addition, this is also consistent with the current guidelines. The American Academy of Neurology published an article in 2011 that pregabalin is an effective treatment and pointed out other drugs that may be effective for DPN treatment, such as venlafaxine and amitriptyline (19). Guidelines from the Federation of European Neurological Societies and the Canadian Pain Society recommend pregabalin as the first-line treatment for neurogenic pain (20, 21). The current guidelines believe that due to the lack of long-term effective evidence and the increasing evidence of serious risks of opioids, especially addiction, abuse, it is not recommended to use opioids to treat chronic pain (19). Also in our survey, only moderate Participants with severe pain and severe pain received opiate therapy (3.03%), indicating that the use of opioids in China has been well managed to relieve DPN pain. Other commonly used analgesics include TCA, anticonvulsants, topical preparations, and serotonin-norepinephrine reuptake inhibitors, which are consistent with the current commonly used analgesics in clinical painful DPN patients (22). In our study, there are still 25.7% of patients with DPN who have an NRS score of 4 or higher without using analgesics. Studies have shown that only 38.6% of diabetic pain patients in France receive appropriate treatment for neuropathic pain (23). A study in Belgium showed that 28% of patients received appropriate neuropathic pain treatment (24). A cross-sectional survey of adult DPN cases in the United States showed that 81.3% of patients had at least one drug for their painful DPN (16). In summary, our research shows that the current pain management of painful DPN in China is still relatively inadequate.

We studied the correlation between NRS pain severity, depression, quality of life, age, duration of diabetes and other factors. The study found that age, duration of diabetes, and duration of DPN are related to the severity of NRS pain, which is consistent with other studies. At the same time in our study, in DPN cases, PHQ-9, GAD-7 and SF-12 score are independently correlated with NRS score. As the NRS score of DPN patients gradually increased, their GAD-7 and PHQ-9 scores also gradually increased. Among diabetic patients who used analgesics, The proportions of GAD-7 and PHQ-9 scores greater than 4 are 91.5% and 32.8%, respectively, indicating that despite the use of analgesics, the risk of depression and anxiety in patients cannot be ignored. The proportion of patients who did not use analgesics with a PHQ-9 score greater than 9 is 44.7%, which also indicates that the current treatment for depression in DPN patients is still incomplete. Secondly, in terms of the SF-12 quality of life score, patients who used analgesics also had lower scores compared with patients who did not use analgesics, which showed that the quality of life of patients who only used analgesics was also significantly lower than that of patients who did not use analgesics.

From free-text responses, there are still quite a few patients who are not satisfied with the effects of analgesics. In addition, since China is still a developing country and most of the patients with DPN are older, the economic burden is still one of the important factors affecting the use of analgesics. We also found that although all patients regard the effect of drugs as the primary consideration for the use of analgesics, there are still big differences in the factors that patients consider analgesics depending on age, education level, and income level. Moreover, most Chinese DPN patients do not pay attention to the addictiveness of analgesics, except for patients with high income and high education level. The above results suggest that in actual clinical treatment, we still need to consider comprehensively and formulate individualized treatment plans according to the patient’s situation when choosing a treatment plan.

This study utilized validated instruments increasing the reliability of our findings. However, our research still has the following limitations. Therefore, it is difficult to make random inferences between health-related characteristics and sociodemographic characteristics. We recruited a homogenous sample, mainly the Han nationality in the Central Plains of China. It is necessary to conduct research on a larger and more diverse sample. Second, we did not use more reliable and quantitative methods to evaluate the diagnosis of DPN, such as nerve conduction studies. Third, due to limited outpatient medical resources, although we excluded patients with known medical diseases that cause non-neurological or mixed pain at the time of enrollment, it is still possible to include some patients with non-neurological or mixed pain in the scope of the investigation. Nonetheless, our research fills a gap in important knowledge about DPN patients’ quality of life, mental and psychological conditions, their use of analgesics, and unmet needs.

## Conclusions

Overall, our cross-sectional study not only provides unique clinical information on the use of analgesics in Chinese patients with painful DPN, but also highlights opportunities for improving management strategies. Our research found that in patients with painful DPN who use analgesics, the quality of life and mental status of the patients are significantly impaired. Therefore, in the practice of clinical management of DPN, improving the quality of life and mental status of patients still cannot be ignored. In addition, there are still a considerable number of patients who meet the conditions for the use of analgesics due to economic burden and other reasons, but they are still not using related drugs, suggesting that the current management level of painful DPN is still insufficient in China.

## Data Availability Statement

The original contributions presented in the study are included in the article/supplementary material. Further inquiries can be directed to the corresponding author.

## Ethics Statement

The studies involving human participants were reviewed and approved by Ethics Committee of Xijing Hospital, Air Force Military Medical University. The patients/participants provided their written informed consent to participate in this study.

## Author Contributions

RC, CZ, HW, and JL were responsible for the collection of questionnaires and data collation. JF was responsible for the design of the subject. JL and JF were responsible for the analysis and summary of the data and writing articles, and JF was responsible for the revision of the manuscript. All authors contributed to the article and approved the submitted version.

## Funding

This research was funded by the National Natural Science Foundation of China (81670736).

## Conflict of Interest

The authors declare that the research was conducted in the absence of any commercial or financial relationships that could be construed as a potential conflict of interest.

## Publisher’s Note

All claims expressed in this article are solely those of the authors and do not necessarily represent those of their affiliated organizations, or those of the publisher, the editors and the reviewers. Any product that may be evaluated in this article, or claim that may be made by its manufacturer, is not guaranteed or endorsed by the publisher.
